# Physicochemical Characteristics, In Vitro Ruminal Digestibility, Bioactive Compounds, and Estimated Methane Production of Wild Floral Species in Goats from the Republic of Malta: A Descriptive Study

**DOI:** 10.3390/vetsci13050427

**Published:** 2026-04-28

**Authors:** Jamie Buttigieg, Emmanuel Sinagra, Everaldo Attard

**Affiliations:** 1 Chemistry Department, Faculty of Science, University of Malta, MSD2080 Msida, Malta; jamie.buttigieg.17@um.edu.mt (J.B.); emmanuel.sinagra@um.edu.mt (E.S.); 2 Institute of Earth Systems, University of Malta, MSD2080 Msida, Malta

**Keywords:** ruminants, forage, wild species, antioxidant activity, plant substrates, polyphenols, sustainability, global warming, chemical composition, fermentation

## Abstract

Pasture plants can contribute to ruminant nutrition and may, depending on composition, influence rumen fermentation and methane production. This study evaluated the nutritional composition, bioactive compounds, and methane production potential of 32 terrestrial plant species commonly foraged by goats in Malta. Dried plant samples were analysed for proximate composition using near-infrared spectroscopy, total polyphenols using the Folin–Ciocalteu assay, antioxidant activity using the DPPH assay, and methane production using an in vitro rumen fermentation system incubated for 48 h, with rumen fluid pooled from three goats (analyses performed in triplicate). Crude protein ranged from 1.16 to 31.97% DM, neutral detergent fibre from 12.29 to 48.89%, and ash from 9.69 to 17.20% across species. Total polyphenolic content varied from 0.07 to 1.30% (*w*/*w*), while antioxidant activity (IC_50_) ranged from 0.37 to 55.9 mg/mL. Methane production after 48 h ranged from 30.39 to 198.26 L CH_4_ kg^−1^, indicating variation in fermentation characteristics among species. These results indicate that *Rumex bucephalophorus* and *Urtica pilulifera* demonstrated relatively high protein or bioactive values and comparatively lower in vitro methane-related parameters under the conditions tested.

## Simple Summary

Goats and other ruminants produce methane during digestion, contributing to greenhouse gas emissions and reducing feed efficiency. This study evaluated 32 wild terrestrial plant species from Malta to determine their nutritional composition, antioxidant activity, and potential influence on methane production in goats. Crude protein levels ranged widely among species, with several plants exceeding 25% dry matter and the highest values approaching 32%. Fibre content also varied substantially, with neutral detergent fibre ranging from approximately 12% to 49%. Polyphenol concentrations ranged from 0.07% to 1.30% (*w*/*w*), while antioxidant activity differed markedly between species, with IC_50_ values from 0.37 to 55.9 mg/mL. In vitro methane production after 48 h ranged from about 30 to 198 L CH_4_ kg^−1^ depending on the plant species. These results demonstrate that several local plants combine favourable protein content, moderate fibre levels, and bioactive compounds that may influence rumen fermentation and methane formation. The findings identify underutilized Maltese plant species that warrant further evaluation as supplementary feed resources for goats, although in vivo studies are needed before their use can be recommended in practice.

## 1. Introduction

This study exclusively focuses on goats. Goats are an important component of livestock systems, particularly in Mediterranean and other low-input production environments, where they efficiently utilise fibrous plant material for milk and meat production. Among ruminant species, goats represent an important component of livestock systems in Mediterranean regions such as Malta, where they are widely reared for dairy production and are well adapted to grazing diverse local vegetation. However, ruminant digestion also produces methane as a by-product of microbial fermentation in the rumen. Methane is a potent greenhouse gas, with a global warming potential significantly higher than that of carbon dioxide; livestock production is considered a major contributor to anthropogenic methane emissions [1]. In addition to its environmental impact, methane formation represents a loss of dietary energy for the animal, typically accounting for between 2 and 12% of gross energy intake [1,2]. Consequently, strategies that improve rumen fermentation efficiency while reducing methane production are of considerable interest for both environmental sustainability and livestock productivity [3].

Diet composition plays a critical role in influencing rumen microbial activity and fermentation pathways. In particular, the nutritional quality of forage, including crude protein content, fibre composition, and digestibility, can affect both animal performance and methane production [4]. Pasture plants and forage species rich in digestible nutrients can enhance feed utilisation and improve fermentation efficiency. Recent studies have examined whether plant species differing in fibre, protein, and secondary metabolites may influence both nutritional value and in vitro methane-related outcomes.

Beyond their nutritional composition, many plants contain bioactive secondary metabolites, particularly phenolic compounds, which can influence rumen microbial populations and fermentation dynamics [5,6,7]. Phenolic compounds such as flavonoids, tannins, and phenolic acids are widely distributed in terrestrial plants and are known for their antioxidant, antimicrobial, and anti-inflammatory properties [5,6,7]. Certain phenolic compounds have been reported to influence rumen microbial communities and, in some cases, are associated with reduced methane formation. Certain phenolic compounds, particularly condensed tannins, can reduce methane production in the rumen by inhibiting methanogenic archaea and protozoa or by limiting hydrogen availability for methanogenesis. When present at moderate concentrations, condensed tannins may mitigate methane emissions without significantly reducing feed digestibility, although excessive levels can impair nutrient utilization. In some cases, methane reductions may instead result from indirect effects on rumen microbial communities or fermentation pathways. In addition, the antioxidant capacity of plant-derived compounds may provide further health benefits for animals by mitigating oxidative stress.

In ruminants such as goats, digestibility and methane production are closely linked because both arise from microbial fermentation in the rumen. In in vitro rumen fermentation experiments, feed samples are incubated with rumen fluid under controlled conditions to simulate ruminal digestion, allowing researchers to measure parameters such as substrate digestibility, total gas production, and methane formation [8]. When rumen microorganisms break down carbohydrates, particularly structural components such as cellulose and hemicellulose, they produce volatile fatty acids (VFAs), carbon dioxide (CO_2_), and hydrogen (H_2_). Methanogenic archaea subsequently use hydrogen and CO_2_ to form methane (CH_4_) [8,9]. Consequently, the extent of feed digestibility influences methane production, because greater microbial degradation of organic matter generally leads to increased fermentation and greater availability of hydrogen for methanogenesis [10]. However, methane output also depends on the dominant fermentation pathways; for example, fermentation that produces acetate releases more hydrogen and is typically associated with higher methane production, whereas propionate formation acts as a hydrogen sink and can reduce methane generation [10]. Thus, measurements obtained from in vitro digestibility and gas production assays provide valuable insights into how different feed substrates influence rumen fermentation patterns and methane emissions [9,10].

The study was conducted in the Republic of Malta, a small island state that is densely populated and located in the central Mediterranean Sea. Malta has a Mediterranean climate, characterised by hot, dry summers and mild, wet winters. The islands are predominantly low-lying limestone plateaus, with limited permanent freshwater resources and a fragmented agricultural landscape. In Mediterranean environments such as Malta, livestock production is often constrained by limited pasture availability and high dependence on imported feed. Nevertheless, the islands host a diverse range of native and naturalised terrestrial plant species that are frequently grazed by animals or occur in areas accessible to livestock. Despite this botanical diversity, relatively little research has examined the nutritional composition and bioactive compound content of Maltese wild flora in the context of ruminant feeding. Characterising these plants may help identify species that justify further evaluation as locally available forage supplements.

Although these species are commonly present in areas accessible to grazing livestock, their nutritional and bioactive properties and their potential influence on rumen fermentation and methane production remain largely unexplored. By characterising the physicochemical properties of these plants, the study seeks to address this knowledge gap by assessing their nutritional and bioactive profiles and exploring whether these characteristics are associated with differences in in vitro methane-related fermentation outcomes. The results can provide preliminary indications of how certain plant species may influence rumen fermentation and methane production under in vitro conditions; however, their implications for animal health and environmental sustainability require confirmation through in vivo studies.

Therefore, this study aimed to characterise the nutritional composition, phenolic content, antioxidant activity, and in vitro rumen fermentation properties of wild plant species consumed by goats in Malta, and to explore how these compositional traits may influence digestibility and methane production.

## 2. Materials and Methods

### 2.1. Feed Resources and Sample Preparation

Plants that are typically foraged by goats were collected to be used and tested as plant species. In this study, the plant materials analysed are occasionally referred to as plant substrates. This terminology is used to reflect their potential application as supplementary components in ruminant diets aimed at improving feed efficiency and reducing methane emissions, although the materials examined consist of naturally occurring terrestrial plant species. A total of 32 terrestrial plants were collected from various valleys around Malta, namely, Wied il-Għasel (Mosta, Malta; N 35° 54′ 46.7806″, E 14° 25′ 34.8647″—11/32), Wied Ħesri (Siġġiewi, Malta; N 35° 50′ 42.62″, E 14° 25′ 55.51″—7/32) and Wied Babu (Żurrieq, Malta; N 35° 49′ 28″, E 14° 27′ 37″—14/32). Malta, an archipelago of islands at the centre of the Mediterranean Sea, characterized by hot, dry summers and mild, wet winters. Rainfall occurs mainly between October and March, while summers are typically warm, sunny, and very dry. The collection was carried out over a one-year period (2023/2024), according to the seasonality of the plant during the flowering phase. The list of the plant species and the proximate analysis results obtained on the dried materials are shown below in Table 1. The aerial parts of all plants were dried in an oven at 40 °C for 48 h, then ground with a blender to pass through a 1 mm mesh sieve and stored until further testing. Methanol extracts were prepared by mixing approximately 0.5 g of ground plant material, with 10 mL of methanol. The samples were vortexed for 30 s, ultrasonicated for 5 min and then transferred to a freezer at −20 °C for 8 h.

### 2.2. Proximate Analysis

Near-infrared (NIR) spectra were obtained using a SpectraStar™ XT NIR spectrophotometer (Unity Scientific, Brookfield, WI, USA). Around 30 g of each plant species was placed in a quartz sample holder, sealed with a gold reflector, and positioned over the 1 cm diameter sample window to maintain close contact and reduce interference from light scattering. For spectral collection, each sample was rotated and scanned three times, resulting in ninety readings per sample, to improve consistency. Absorbance spectra were collected across wavelengths from 1400 to 2500 nm. Proximate analyses were conducted in triplicate on the samples. The measured parameters included dry matter (DM), crude protein (CP), ether extract (EE), neutral detergent fibre (NDF), acid detergent fibre (ADF), and total ash [11]. The non-fibre carbohydrate (NFC) was calculated as in Equation (1). Energy (kcal/100 g) was estimated from proximate composition using Equation (2), expressed on a dry matter basis.

(1) NFC (%)=100−(CP+NDF+EE+Ash)

(2) Energy (kcal/100 g)=(4×CP)+(9×EE)+(4×NFC)

### 2.3. Inoculum Source

Ruminal fluid was obtained from the Government Abattoir from culled goats that had been maintained under controlled feeding conditions throughout lactation (commercial feed supplemented with flakes and cereals, crushed corn feed, and dry goat feed; a ratio of approximately 60:40, forage to concentrate) and transported from nearby farms. Samples were collected from three goats (1 L per animal), pooled, and delivered to the laboratory within 30 min of slaughter. Collection was carried out in vacuum-sealed flasks pre-flushed with CO_2_. The rumen contents from the three goats were homogenized using a sterilized blender that had also been flushed with CO_2_. The homogenate was passed through sterile muslin. To allow cryopreservation at −80 °C, dimethyl sulfoxide (DMSO, 5%) was added to the rumen filtrate. The mixture was then portioned (4 mL) into sterile 15 mL centrifuge tubes pretreated with CO_2_. These tubes were then placed in a −20 °C freezer in an isopropanol bath for one hour to achieve a controlled cooling rate of 1 °C per minute. Afterwards, they were transferred to a −80 °C freezer for another one hour while still kept in an isopropanol bath. Once the hour elapsed, the tubes containing the inoculum were removed from the isopropanol bath and stored at −80 °C [11]. Microbial viability was assessed at the preparation time to determine consistency in the subsequent resuscitation of the frozen cultures.

### 2.4. In Vitro Gas Production

In vitro fermentations were carried out in 250 mL gas-tight jars fitted with a side arm and valve for gas collection. Menke’s medium was prepared and maintained at 39 °C in a water bath under continuous CO_2_ flushing [11]. For each run, five jars were prepared, each containing 78 mL of buffer, 15 mL of ruminal fluid and 0.5 g of randomly assigned plant material (three replicates per feed and one control). Cryopreserved ruminal fluid in DMSO was thawed in the water bath and used as the inoculum, while blanks consisted only of buffer and rumen fluid. The jars were then incubated at 39 °C for 48 h. Each plant substrate was tested in triplicate; therefore, the plant substrate was added in three different jars. Triplicate measurements were used as the minimum level of replication to improve reliability. It should be noted that the inoculum used in all replicates was from the homogenised ruminal fluid. Furthermore, it was confirmed that the jar positions were random, therefore avoiding any position biases.

Measurements of pH (Thermo Scientific Orion 4-Star, Thermo Fisher Scientific Inc., Waltham, MA, USA) were carried out at the start and at the end of each incubation. At time 0, CO_2_ saturation and medium pH were verified by the resazurin indicator, which shifted in colour from purple to pink or colourless [11].

### 2.5. Methane Determinations

Methane concentration in the jars containing the medium, inoculum and plant substrate was measured at 0, 6, 24, and 48 h, through the gas valve attached to the side arm of the jars. A handheld infrared methane detector (EIRAA, P.R.C.) was used for the measurements. The side-arm outlet was opened, and readings were taken after 5 s [12]. Equation (3) was used to calculate methane production at the time intervals.

(3) $$Methane\;(L\;{CH}_{4}/kg)=\frac{\left(\frac{\%{CH}_{4}}{100})\times V_{headspace}\;(L\right)}{m_{sample}\;(kg)}$$

Methane production across time was modelled using a non-linear regression based on the first-order kinetics model described by Hashimoto [13] (Equation (4)).

(4) $${BMP}_{t}=B_{0}(1-{exp}^{-kt})$$

In this model, BMP_t_ (BioMethane Production) denotes the cumulative CH_4_ yield (L CH_4_ kg VS^−1^) at a given time *t* (days), B_0_ represents the maximum CH_4_ yield (L CH_4_ kg VS^−1^), and *k* is the BMP rate constant (day^−1^), which is substrate-dependent and reflects the time needed to reach a fraction of B_0_.

### 2.6. Total Polyphenolic Content

The total phenolic content of the methanolic extracts was determined using the Folin–Ciocalteu colorimetric method, with gallic acid as the standard. A stock solution of gallic acid was prepared (960 μg/mL). Serial dilutions were then performed to obtain standard concentrations of 480, 240, 120, and 60 μg/mL, while distilled water served as the 0 μg/mL blank. The Folin–Ciocalteu reagent was prepared as a 10% (*v*/*v*) solution, and sodium carbonate was prepared as a 1 M solution.

In a 96-well microplate, 10 μL of each standard or methanolic extract was transferred in triplicate. Subsequently, 100 μL of 10% Folin–Ciocalteu reagent was added to each well, followed by 80 μL of 1 M sodium carbonate solution. The plate was incubated at room temperature in the dark for 20 min. Absorbance was then measured at 750 nm using a microplate reader (SpectraMAX 340PC, Molecular Devices Corporation, San Jose, CA, USA). The results were expressed as gallic acid equivalents (GAE) [14].

### 2.7. Antioxidant Activity

The antioxidant activity of samples was evaluated using the DPPH radical scavenging assay in a 96-well microplate format. A 0.2 M DPPH solution was prepared in methanol, and test samples were serially diluted across the plate. For each sample, wells containing sample alone (blank) and sample plus DPPH were included, alongside a DPPH control. Following the addition of DPPH, plates were covered, protected from light, and incubated at room temperature for 30 min. Absorbance was then measured at 517 nm using a microplate reader. Radical scavenging activity (%) was calculated relative to the control, and calibration curves were used to determine both the absolute amount of DPPH reduced and the EC_50_ values, defined as the concentration of sample required to quench 50% of the initial DPPH radicals. All measurements were performed in triplicate [14].

### 2.8. Statistical Analysis

It should be noted that throughout the study, various data tables and figures are presented as means and feature ± values to denote the standard deviation (SD). The acceptance was kept at <5% CV for excellent analytical precision. However, in some cases a 5–20% CV was accepted, which is acceptable for biological data. Statistical analyses were conducted using repeated measures ANOVA in SPSS software (version 26.0, SPSS Inc., Chicago, IL, USA). Tukey’s post hoc test was applied to identify significant differences in methane production at the end point. Two-way ANOVA was used to determine Plant, Time, and Plant × Time interactions, all using methane measurements (6 h, 24 h, 48 h with replicates). Principal Component Analysis (PCA) was carried out on all proximate feed parameters using XLSTAT (Microsoft, version 19.4.46756, SAS Institute Inc., Marlow, Buckinghamshire, UK) to examine correlations or differences between the plant species. A significance threshold of *p* < 0.05 was applied to all analyses.

## 3. Results

### 3.1. Plant Species Composition Assessment

The proximate analyses of individual plant species are presented in Table 1. The plant species varied in composition. Dry matter content ranged from 100.9 ± 1.49% in plant number 41-As, known as *Galactites tomentosa* (Mediterranean thistle), to 83.72 ± 1.77% in plant number 18-Po, known as *Rumex bucephalophorus* (Red Dock). Ash content ranged from 17.2 ± 0.37% in plant number 18-Po, known as *Rumex bucephalophorus* (Red Dock), to 9.69% in plant number 41-As, known as *Galactites tomentosa* (Mediterranean thistle). Crude Protein content ranged from 27.31 ± 3.13% in plant number 68-As, known as *Matricaria chamomilla* (Scented Mayweed), to 1.157 ± 3.01% in plant number 41-As, *Galactites tomentosa* (Mediterranean Thistle). Ether extract content ranged from 9.457 ± 1.16% in *Galactites tomentosa* (Mediterranean Thistle) to 0% in many of the plant species. NDF content ranged from 48.89 ± 0.38% in plant number 40-Xa, *Asphodelus ramosus* (Branched Asphodel), to 12.29 ± 4.46% in plant number 27-Ma, known as *Malva sylvestris* (Common Mallow). ADF content ranged from 13.47 ± 7.02% in plant number 22-As, known as *Sonchus asper* (Prickly Sow Thistle), to 31.94 ± 8.32% in plant number 41-As, known as *Galactites tomentosa* (Mediterranean Thistle).

### 3.2. Folin–Ciocalteu (FC) Results

From Table 2, it can be observed that the plant with the lowest PolyP (% *w*/*w*) value is plant number 47-La, known as *Prasium majus* (White Hedge-Nettle), at a value of 0.074 ± 0.00, while the highest PolyP (% *w*/*w*) value is plant number 30-Br, known as *Brassica rapa* subsp. *sylvestris* (Wild Turnip), at a value of 1.301 ± 0.03.

### 3.3. Methane Production

Table 3 presents methane production over the 48 h period for all plant species. Methane production varied significantly by plant substrate, reflecting differences in fermentability and methanogenic potential. The highest producers (category “a,” e.g., 20, 39, 74, 28) generated the greatest methane volumes (≈173–198 L CH_4_/kg after 48 h), indicating highly fermentable substrates. Conversely, a large group (category “c,” e.g., 15, 18, 19, 27, 30, 31, 33, 36, 44, 46, 63, 64, 68, 78) consistently produced low methane (<72 L CH_4_/kg), suggesting lower degradability or inhibition (e.g., secondary metabolites). Intermediate producers (“b”) showed moderate, variable outputs. Kinetic data showed that potential (B_0_) and rate (k) were not always linked (e.g., high B_0_ but low k for 14, 43; high k but moderate B_0_ for 28, 47). Evaluating plant substrates thus requires considering both the extent (B_0_) and rate (k) of fermentation, as they determine total methane production and its temporal dynamics.

**Figure 1:**
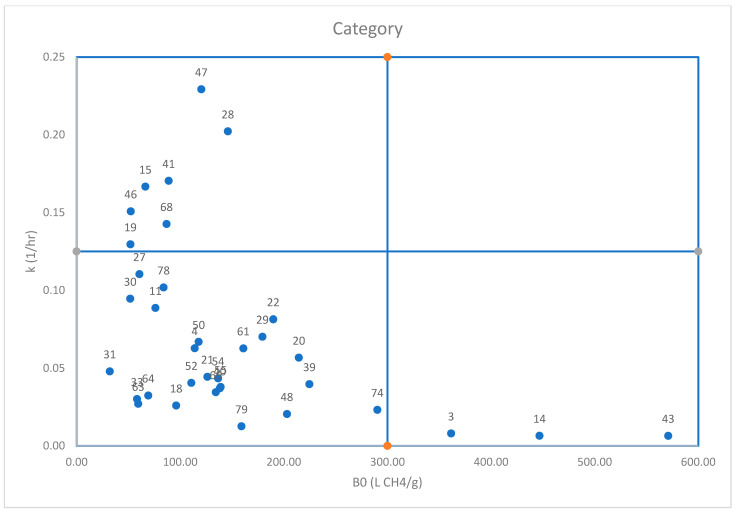
The relationship between k (1/h) and B_0_ (L CH_4_/g) for the plant species.

The relationship between k (1/h) and B_0_ (LCH_4_/g) for the plant species is represented in Figure 1. The relationship showed no clear linear correlation, indicating a decoupling between the extent and kinetics of ruminal fermentation. Most plant species clustered in the low B_0_–low k region, suggesting limited fermentability and reduced methanogenic potential. In contrast, a few additives exhibited high B_0_ but low k, reflecting a high methane production potential occurring at a slower rate. Only a small number of samples combined relatively high k with moderate B_0_, indicative of rapidly fermentable substrates.

## 4. Discussion

The present study combined proximate analysis, polyphenolic content, antioxidant activity, and in vitro methane-related measurements to compare the nutritional and bioactive profiles of the plant species. The nutritional composition of the studied species, particularly the balance of dry matter (DM), crude protein (CP), ether extract (EE), neutral detergent fibre (NDF), and acid detergent fibre (ADF), provides comparative compositional information relevant to their possible use in ruminant feeding. The NIR analysis indicates diversity in the physicochemical composition of the analysed plants, with each species demonstrating distinct compositional traits that may be relevant to in vitro fermentation behaviour.

Some bromatological and kinetic parameters showed unusually high variability or values outside the expected biological range. These results should therefore be interpreted cautiously, as they may partly reflect analytical uncertainty associated with NIR-predicted compositional parameters, variability inherent to in vitro rumen fermentation systems, or sensitivity of nonlinear kinetic models to small fluctuations in gas production measurements. Consequently, individual extreme values should not be interpreted as definitive physiological differences between plant species but rather as indicative trends within the methodological limitations of the experimental approach.

Ash represents the inorganic portion of the plant, indicating the mineral content and the potential nutritive value of soil at the time of sampling [15]. Comparable findings on ash content across plant families have been reported in the literature. High ash in *Rumex bucephalophorus* (83.72 ± 1.77%) suggests a high mineral content, potentially enhancing its value as a livestock nutritional supplement [16]. However, excessive ash can reduce digestible nutrients [17]. Ash content correlates with essential minerals. Asteraceae (As) showed the widest ash range (9.69–16.39%), while Xanthorrhoeaceae (Xa) had the highest mean (15.46%). ANOVA found no statistically significant difference between plant families. The literature supports varying ash content across families. Asteraceae species showed a narrower range (~5.7–11.8% DM) than observed here [18]. Conversely, Aloe (Asphodelaceae/Xanthorrhoeaceae) often reports higher ash (e.g., 19.5% DM) [19], consistent with the elevated mean for this family. Ash content differed significantly among species, going beyond factors like growing season or plant part. However, in some cases these factors override taxonomic grouping in ash content variance [20,21]. Furthermore, intra-family variation, even in *Aloe*, reinforces that taxonomic level alone may not fully explain ash patterns [22].

Other studies, like one examining 156 species across 35 botanical families, similarly found variation in dry matter content but no consistently statistically significant family-level differences [23]. This aligns with the present findings: while Caprifoliaceae had the lowest minimum dry matter (70.64%) and Asteraceae the highest maximum (100%), dry matter content varied moderately among species. This suggests dry matter content is more influenced by species- or environment-specific factors than by family membership [23].

Protein is vital for ruminant growth, reproduction, lactation, and metabolism [24,25]. The high nutritional composition of these plants is valuable for optimizing livestock feed, especially in dairy production, where high protein is crucial during early lactation to meet increased metabolic demands for milk production [25]. Species like *Urtica pilulifera* (Roman Nettle) (63-Ur), with crude protein (CP) exceeding 25%, are suitable for this purpose, potentially enhancing milk yield. The plant family with the highest maximum CP was Caprifoliaceae (Ca) at 33.11%, while the lowest minimum was Boraginaceae (Bo) at 20.68%. Lamiaceae (La) had the highest mean CP (29.33%), making it a strong compromise choice. However, CP includes non-protein nitrogen (e.g., urea, ammonia), which can affect amino acid availability [26]. Crude protein showed moderate variability across species but relatively limited statistical separation.

Forage crude protein (CP) is highly variable (2–36%) across species and functional groups, with legumes generally higher than grasses or herbs [27]. Field studies often find significant inter-specific differences in CP, but weaker or inconsistent effects at higher taxonomic levels (e.g., families) due to within-family variation and limited ANOVA power [28]. Thus, a family’s high mean or maximum in one dataset might not translate to significant family-level differences, as species level and environmental variation can dominate [29]. Targeted studies on Lamiaceae herbs and medicinal rangeland species report species-specific, generally moderate CP (e.g., 8–12% DM), suggesting that labelling the entire Lamiaceae family as high-protein depends heavily on the specific species composition sampled.

Ether extract (EE) measures crude fat (lipids, waxes, etc.) [30]. EE values were generally low across species, with *Galactites tomentosa* (sample 41-As) having the highest EE (9.457 ± 1.16%). These low levels suggest limited energy contribution and align with the typical 2–3% fat range for forage species [31]. While not ideal for high-lipid applications, they may contribute minor essential fatty acids [32], as seen in *Sonchus asper* (sample 22, 2.443 ± 0.79%) and *Plantago lagopus* (sample 15, 2.947 ± 0.42%). The generally low EE values indicate that lipids were a minor compositional component in most samples. Urticaceae (Ur) generally showed negligible EE, while Asteraceae (As) had high values, including the maximum from *G. tomentosa*. Conversely, Geraniaceae (Ge) had the highest mean EE (4.19%), making it the most suitable family for increasing fat. Although one Urticaceae sample recorded the lowest EE (0.2467 ± 0.22), the family’s mean remained high (4.15%) due to *Urtica pilulifera* (sample 63), which had the second-highest individual EE (8.05 ± 1.27%). Moderate EE may, depending on lipid composition, influence rumen fermentation and methane production [33]; however, lipid composition was not characterised in the present study. Differences detected among some species may reflect variation in plant secondary metabolites or seed-derived lipids.

Dietary fibre (NDF and ADF) is essential for ruminant health, supporting rumination and gut stability, though its effect on milk fat is minor [34,35]. While high-fibre plants are less digestible, they suit high-fibre diets, whereas low-fibre plants offer greater digestibility. High NDF/ADF diets can increase methane emissions due to slower fermentation [36]. The balance between fibre and protein can influence rumen fermentation and nutrient use, although feed efficiency was not assessed in the present study.

Plant fibre content varied widely: NDF ranged from 10.38% (*Asphodelus fistulosus*) to 54.84% (*Avena barbata*). High NDF, like in Avena barbata (54.84%) and *Rumex bucephalophorus* (51.85%), reflects structural carbohydrates, aiding rumen activity but potentially limiting intake and energy [37]. The Xanthorrhoeaceae had the lowest minimum NDF (11.69%), Asteraceae the highest maximum (48.89%), and Apiaceae the highest mean (32.37%). NDF values varied widely among species, indicating differences in total structural carbohydrates. ADF, representing less digestible fractions (lignin, cellulose), ranged from 14.24% (*Asphodelus fistulosus*) to 39.14% (Lonicera implexa). Low ADF in *Asphodelus fistulosus* indicates high digestibility and energy. High ADF species like *Lonicera implexa* offer less digestible energy, suitable for maintenance diets, while moderate ADF plants like *Erica multiflora* (24.3%) and *Urtica pilulifera* (24.28%) can be supplementary fibre sources. Caprifoliaceae had the lowest minimum ADF (13.04%), Asteraceae the highest maximum (32.55%), and Apiaceae the highest mean (25.49%). ADF values also differed among species, reflecting variability in cellulose and lignin fractions.

To optimize dairy goat performance, a strategic balance of high-protein and high-fibre plants is recommended, tailored to the lactation stage [38]. From a nutritional standpoint, balancing protein and fibre is generally relevant in dairy goat feeding; however, the present study did not assess animal performance outcomes [38]. To this end, the energy levels of each plant family were also analysed. The Asteraceae (As) group was shown to have the lowest minimums at 337.8 kcal/100 g; however, it also had the highest maximums at 408.5 kcal/100 g. Although Boraginaceae showed the highest mean value in this dataset, no statistically significant differences were detected among plant families. Nevertheless, ANOVA showed that there was not a statistically significant difference between any of the plant family groups.

Despite its non-specificity, the robust FC assay is a popular method for estimating the antioxidant capacity of phenolic-rich samples. Polyphenolic content differed significantly among plant species (*p* < 0.001), indicating marked variability in their secondary metabolite profiles (Table 2). *Erica mutiflora* (Ericaceae) was the highest performer (1.31% *w*/*w*). This elevated phenolic content suggests greater antioxidant-related potential in vitro; however, antimicrobial or anti-inflammatory effects were not assessed in this study. High phenolic levels, especially tannins, must be balanced as they can negatively affect feed intake and nutrient digestibility. *Solanum nigrum* (Solanaceae) had the lowest overall polyphenols (0.074% *w*/*w*). This indicates a limited antioxidant contribution, suggesting a less pronounced direct impact on rumen oxidative stress and microbial activity as well as improved milk phenolic content [39]. These plants may be suitable when moderating phenolic load is necessary, serving as a neutral nutritional base to complement high-phenolic feeds. These findings may be relevant to hypotheses concerning methane mitigation, but their biological significance requires confirmation in vivo. Polyphenols are known to modulate rumen fermentation by inhibiting protozoa and methanogens, thus reducing enteric methane emissions [40]. For example, the sugarcane polyphenol-rich product Polygain™ reduced methane emissions by approximately 24% in dairy cattle [41]. Whether similar effects would occur in goats fed these plant species remains unknown and would need direct in vivo evaluation.

Table 2 also shows the IC_50_ values determined through DPPH testing. IC_50_ values showed significant differences (*p* < 0.001), with higher polyphenolic content generally associated with lower IC_50_ values, reflecting stronger antioxidant activity. DPPH radical scavenging assay IC_50_ values varied significantly, from 0.37 ± 0.09 mg/mL (*Rumex bucephalophorus*, 18-Po) to 55.9 ± 5.73 mg/mL (*Fumaria capreolata*, 33-Fu), with a mean of 6.84 mg/mL. Lower IC_50_ indicates stronger antioxidant capacity. *Rumex bucephalophorus* (18-Po) showed the highest efficiency (lowest IC_50_), suggesting potent antioxidant constituents, comparable to *Centaurea nicaeensis* (66-As, 0.433 ± 0.09 mg/mL) and *Teucrium fruticans* (44-La, 0.633 ± 0.09 mg/mL). Conversely, *Fumaria capreolata* (33-Fu) exhibited the weakest activity (highest IC_50_), possibly due to lower or less reactive phenolic compounds. Other weaker species included *Calendula arvensis* (20-As, 22.7 ± 2.17 mg/mL) and *Gladiolus italicus* (36-Ir, 11.5 ± 2.33 mg/mL). This broad range highlights significant interspecies variability. *Rumex bucephalophorus*, *Centaurea nicaeensis*, and *Teucrium fruticans* are promising candidates for further analysis of their potent radical-scavenging compounds.

In this research, fermentation trials were carried out to evaluate methane generation during ruminal digestion in goats. It is well established that the composition of feed rations influences the microbial communities present in ruminal fluid [42]. To explore this, the nutritional characteristics of each plant species were assessed individually to determine which factors might impact the fermentation process. Substrate type, retention time in the rumen, and overall feeding efficiency are among the key drivers of methane output [43].

As shown in Table 3, plant species were tested, each displaying unique methane production profiles at 6, 24, and 48 h, highlighting differences in their temporal dynamics. Two-way ANOVA revealed significant effects of plant species (F_46_ = 21.80, *p* < 0.001), incubation time (F_2_ = 475.36, *p* < 0.001), and their interaction (F_92_ = 7.29, *p* < 0.001) on methane production, indicating that both substrate type and fermentation time significantly influenced methane generation dynamics. The Hashimoto first-order kinetic model was applied, in which B_0_ (L CH_4_/g) denotes the maximum methane yield obtainable per gram of substrate, while k (1/h) reflects the degradation rate and conversion speed of the substrate to methane [13]. The results enabled the classification of the tested plant species into four categories, as summarized in Table 4. The thresholds used for this classification were as follows: B_0_ values of ≥117.77 L CH_4_/g were considered high and <117.77 L CH_4_/g was considered moderate, while k values of ≥0.0567 1/h were categorized as high and <0.0567 1/h as low to moderate.

B_0_ represents the highest possible methane yield from a substrate when given adequate time for complete degradation under optimal conditions. A large B_0_ value, such as 571 L CH_4_/g for *Foeniculum vulgare* subsp. *vulgare* (fennel), suggests that the substrate has strong methane-generating potential, likely due to its richness in organic matter, which can be efficiently converted into biogas. Because the study was designed primarily as a comparative screening of plant species under standardized in vitro conditions, individual outlying kinetic values likely reflect methodological sensitivity rather than biologically implausible fermentation behaviour, and should not be overinterpreted in isolation. On the other hand, *Asphodelus ramosus* (branched asphodel) or *Avena barbata* (Barbed Oat) break down more quickly, as indicated by their elevated k values, but yield less methane overall. This lower potential may stem from a reduced amount of degradable organic matter or from chemical characteristics that restrict methane release, particularly in their dried form, as shown through similar studies [44].

The k parameter further describes how quickly a substrate is decomposed into methane. A high k (e.g., 10.71 1/h for *Asphodelus ramosus*) signals rapid degradation and conversion, whereas a low k (e.g., 0.01 1/h for *Foeniculum vulgare*) reflects a much slower process that requires extended digestion time to reach its full methane output. This delayed breakdown often relates to structural complexity or a high lignin content, as seen in fennel, which resists microbial breakdown.

When selecting additives, both substrate type and digestion rate should be considered. To limit methane emissions, it is preferable to reduce the inclusion of plant species with high B_0_ values. However, degradation speed (k) is also a decisive factor, since it influences how efficiently and how quickly methane is released. Plant species such as plant numbers 43-As and 14-Fa show high B_0_ but low k values, making them less favourable where lower methane-related outputs are desired. Conversely, additive numbers 18-Po, 20-As, 31-Br, 33-Fu, 63-Ur, and 64-Ur demonstrated moderate B_0_ values coupled with low k rates, highlighting their potential relevance for future studies on lower-emission feeding approaches.

**Figure 2:**
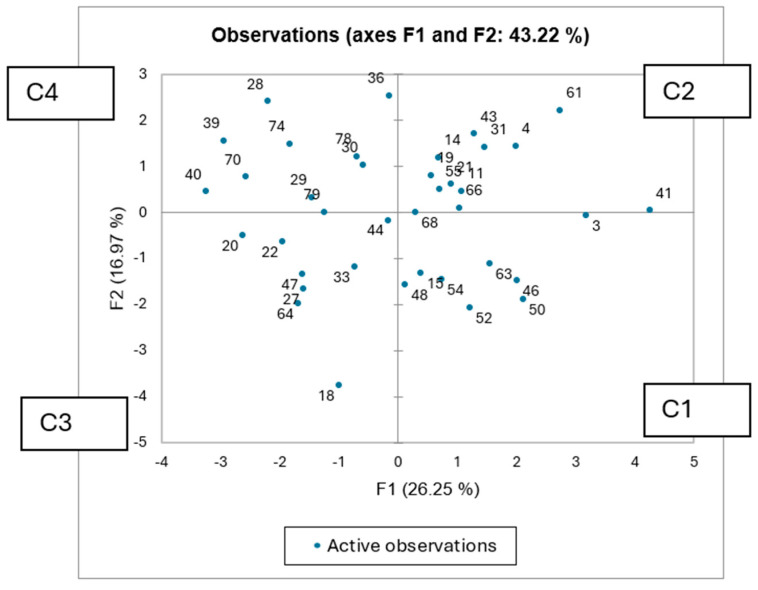
Observation plot (PCA) for the plant species based on all the parameters tested. C1 to C4 refer to clusters distinguished via AHC analysis.

**Figure 3:**
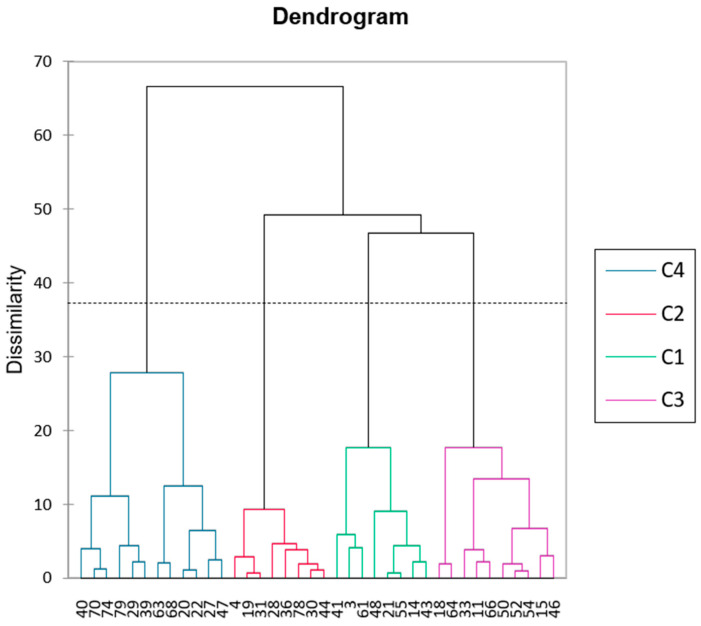
AHC analysis for the plant species under study.

PCA (Figure 2) of the nutritional/functional parameters (excluding five inconsistent samples) showed the first two principal components explaining 42.61% of the total variance (F1: 25.68%, F2: 16.93%). Table 5 summarises the eigenvectors for the twelve parameters as well as the eigenvalues and variability of the first two principal components. F1 separates species based on energy/compositional parameters (positive) versus fibre-related characteristics (negative), while F2 distinguishes species by phenolic content and antioxidant variables. This highlights a gradient from nutritionally dense species to those with higher fibre or bioactive compounds. AHC analysis (Figure 3) identified four clusters (C1–C4 in Figure 2), independent of taxonomic classification. Table 6 represents the categorisation of the plant species within the four clusters: C1: Balanced nutritional profiles, moderate digestibility, and detectable bioactive compounds (e.g., *T. fruticans*, *P. lagopus*); C2: Species with comparatively higher digestibility and moderate antioxidant capacity (e.g., *R. picroides*, *G. coronaria*); C3: High crude protein and higher antioxidant activity (e.g., *R. bucephalophorus*, *P. judaica*); C4: Lower antioxidant potential and lower digestibility, associated with fibrous material and distinct bioactive profiles (e.g., *A. ramosus*, *A. tortuosum*).

## 5. Conclusions

Among the plant additives tested, *Rumex bucephalophorus* (18-Po) and *Urtica pilulifera* (63-Ur) were among the more favourable species due to their combined nutritional composition, antioxidant activity, and methane mitigation potential. *Rumex bucephalophorus* showed high mineral content (ash; 17.20 ± 0.37%) and strong antioxidant activity (IC_50_ 0.37 ± 0.08 mg/mL), while *Urtica pilulifera* exhibited high crude protein (>25%) and elevated ether extract (8.05 ± 1.27%), consistent with favourable in vitro fermentation characteristics and possible effects on methane-related fermentation. *Teucrium fruticans* (44-La) and *Centaurea nicaeensis* (66-As) demonstrated low IC_50_ values (0.633 ± 0.09 and 0.433 ± 0.09 mg/mL), and *Erica multiflora* (31-Er) had the highest polyphenolic content (1.31% *w*/*w*), indicating strong antioxidant potential. Digestibility and fermentation results indicated that *Rumex bucephalophorus* and *Urtica pilulifera* showed moderate methane potential (B_0_) and low degradation rates (k) under the in vitro conditions tested, indicating comparatively moderate methane-related fermentation profiles.

These findings identify plant species with comparatively favourable nutritional profiles, antioxidant-related properties, and in vitro methane-related characteristics that may warrant further evaluation for use in goat feeding systems. However, because the study was conducted in vitro, any implications for animal performance, health, or productivity remain uncertain and require confirmation through in vivo trials.

## Figures and Tables

**Table 1:** Chemical composition (%) of the dried individual plant species.

Number Identifier and Plant Family	Latin Name (Common Name)	DM	ASH	CP	EE	NDF	ADF	NFC	Energy (kcal/100 g)
Herbaceous forbs									
4-Bo	*Borago officinalis* (Common Borage)	86.68 ± 2.59 ^a^	9.99 ± 0.55 ^b^	20.68 ± 3.63 ^a^	1.52 ± 0.70 ^b^	26.80 ± 2.80 ^b^	21.82 ± 4.52 ^a^	46.01 ± 5.37 ^a^	363.7 ± 4.6 ^a^
15-Pl	*Plantago lagopus* (Mediterranean Plantain)	86.08 ± 4.04 ^b^	12.73 ± 0.63 ^b^	27.98 ± 7.26 ^a^	2.95 ± 0.42 ^b^	38.43 ± 6.71 ^a^	20.58 ± 2.00 ^a^	30.88 ± 0.26 ^a^	347.0 ± 13.1 ^b^
19-As	*Sonchus oleraceus* (Smooth Sow Thistle)	86.21 ± 1.70 ^b^	13.62 ± 0.87 ^a^	21.09 ± 2.58 ^a^	1.37 ± 1.37 ^b^	26.00 ± 5.57 ^b^	15.58 ± 4.86 ^a^	30.25 ± 2.62 ^a^	352.4 ± 8.87 ^b^
21-As	*Reichardia picroides* (Common Reichardia)	86.83 ± 1.47 ^a^	13.77 ± 0.05 ^a^	21.63 ± 3.37 ^a^	1.64 ± 0.92 ^b^	26.25 ± 2.34 ^b^	19.47 ± 0.65 ^a^	36.72 ± 0.08 ^a^	353.1 ± 4.5 ^b^
22-As	*Sonchus asper* (Prickly Sow Thistle)	86.34 ± 3.00 ^b^	16.39 ± 0.80 ^a^	27.81 ± 3.09 ^a^	2.443 ± 0.79 ^b^	24.30 ± 1.70 ^b^	13.47 ± 6.89 ^a^	36.62 ± 5.50 ^a^	337.8 ± 12.4 ^a^
27-Ma	*Malva sylvestris* (Common Mallow)	87.21 ± 2.46 ^a^	14.68 ± 0.07 ^a^	30.00 ± 3.37 ^a^	0.83 ± 0.83 ^b^	29.05 ± 2.30 ^a^	19.48 ± 0.50 ^a^	26.44 ± 0.32 ^b^	340.5 ± 6.4 ^b^
28-Ra	*Nigella damascena* (Love-in-a-mist)	83.85 ± 0.13 ^b^	12.61 ± 1.45 ^b^	28.60 ± 4.23 ^a^	0.69 ± 0.62 ^b^	23.49 ± 2.56 ^b^	12.31 ± 6.18 ^a^	46.81 ± 8.99 ^a^	348.5 ± 11.2 ^a^
29-Ra	*Adonis microcarpa* (Pheasant’s Eye)	86.94 ± 2.82 ^a^	13.90 ± 0.25 ^a^	28.96 ± 4.66 ^a^	0.86 ± 0.85 ^b^	23.99 ± 2.56 ^b^	23.82 ± 1.53 ^a^	33.88 ± 1.20 ^a^	343.3 ± 7.9 ^a^
30-Br	*Brassica rapa subsp. sylvestris* (Wild Turnip)	83.86 ± 1.05 ^b^	13.89 ± 0.17 ^a^	25.91 ± 2.51 ^a^	1.55 ± 0.81 ^b^	27.72 ± 2.15 ^b^	19.00 ± 0.99 ^a^	36.78 ± 0.53 ^a^	352.2 ± 4.8 ^a^
54-Ap	*Daucus carota* (Wild Carrot)	86.58 ± 2.27 ^a^	13.36 ± 0.42 ^b^	25.69 ± 4.25 ^a^	0.99 ± 0.92 ^b^	28.00 ± 7.28 ^a^	27.42 ± 1.74 ^a^	29.42 ± 0.37 ^a^	347.1 ± 8.3 ^b^
55-Ac	*Acanthus mollis* (Bear’s Breech)	88.44 ± 4.90 ^a^	13.71 ± 0.34 ^a^	21.79 ± 10.08 ^a^	1.73 ± 1.51 ^b^	29.08 ± 4.68 ^a^	26.20 ± 6.01 ^a^	36.46 ± 0.29 ^a^	345.3 ± 10.1 ^b^
Mineral-rich wild herbs									
18-Po	*Rumex bucephalophorus* (Red Dock)	83.72 ± 1.77 ^b^	17.20 ± 0.37	25.30 ± 4.98 ^a^	0.66 ± 0.31 ^b^	33.68 ± 1.92 ^a^	29.8 ± 3.43 ^a^	19.99 ± 3.10 ^b^	326.6 ± 3.74 ^b^
63-Ur	*Urtica pilulifera* (Roman Nettle)	93.03 ± 1.54 ^a^	14.42 ± 0.17 ^a^	29.33 ± 1.95 ^a^	8.05 ± 1.27 ^a^	22.3 ± 5.07 ^b^	25.29 ± 0.51 ^a^	31.54 ± 0.14 ^a^	382.6 ± 6.97 ^a^
64-Ur	*Parietaria judaica* (Pellitory-of-the-wall)	84.91 ± 0.51 ^b^	15.57 ± 0.26 ^a^	27.47 ± 0.30 ^a^	0.2467 ± 0.22 ^b^	29.17 ± 1.14 ^b^	20.99 ± 0.89 ^a^	34.4 ± 4.89 ^a^	338.9 ± 0.15 ^a^
Leguminous forages									
14-Fa	*Scorpiurus muricatus* (Many-flowered Scorpiurus)	88.25 ± 2.32 ^a^	12.76 ± 0.20 ^b^	25.35 ± 4.30 ^a^	1.073 ± 1.41 ^b^	28.83 ± 5.61 ^b^	20.73 ± 1.19 ^a^	35.72 ± 2.15 ^a^	354.3 ± 7.81 ^a^
Fibre-rich structural species									
36-Ir	*Gladiolus italicus* (Field Gladiolus)	86.12 ± 2.52 ^b^	12.5 ± 0.66 ^a^	24.94 ± 6.05 ^a^	1.13 ± 1.36 ^b^	44.78 ± 5.03 ^a^	18.46 ± 0.59 ^a^	44.28 ± 4.05 ^a^	351.2 ± 10.45 ^a^
39-Xa	*Asphodelus fistulosus* (Onion Weed)	85.89 ± 2.09 ^a^	15.66 ± 0.12 ^a^	27.47 ± 4.32 ^a^	0.89 ± 1.20 ^b^	13.62 ± 1.05 ^b^	16.21 ± 1.20 ^a^	45.33 ± 3.84 ^a^	336.6 ± 6.99 ^a^
40-Xa	*Asphodelus ramosus* (Branched Asphodel)	85.21 ± 2.23 ^a^	15.26 ± 0.23 ^a^	28.43 ± 4.68 ^a^	0.8467 ± 1.34 ^b^	48.89 ± 0.38 ^a^	17.49 ± 0.27 ^a^	43.19 ± 4.27 ^a^	336.4 ± 8.01 ^a^
48-So	*Solanum nigrum* (Black Nightshade)	88.27 ± 2.49 ^a^	15.78 ± 0.32 ^a^	26.39 ± 4.76 ^a^	3.945 ± 0.55 ^b^	41.6 ± 12.80 ^a^	16.98 ± 2.00 ^a^	31.94 ± 4.04 ^a^	348.1 ± 8.82 ^a^
Mixed functional Mediterranean rangeland species									
20-As	*Calendula arvensis* (Field Marigold)	84.25 ± 1.84 ^b^	15.63 ± 0.37 ^a^	29.86 ± 6.35 ^a^	1.177 ± 1.53 ^b^	26.25 ± 2.34 ^b^	16.81 ± 0.99 ^a^	27.33 ± 0.40 ^a^	343.4 ± 9.11 ^a^
31-Er	*Erica multiflora* (Mediterranean Heath)	86.62 ± 3.00 ^a^	10.36 ± 0.40 ^b^	26.01 ± 6.68 ^a^	2.183 ± 2.83 ^b^	27.3 ± 2.37 ^a^	22.52 ± 0.91 ^a^	33.72 ± 2.13 ^a^	369.5 ± 15.73 ^a^
33-Fu	*Fumaria capreolata* (White Ramping Fumitory)	86.12 ± 2.79 ^b^	13.65 ± 0.37 ^a^	29.1 ± 5.18 ^a^	0.8667 ± 1.58 ^b^	22.62 ± 7.08 ^b^	23.36 ± 0.81 ^a^	30.75 ± 1.60 ^a^	341.4 ± 9.47 ^b^
41-As	*Galactites tomentosa* (Mediterranean Thistle)	99.56 ± 0.44 ^a^	9.693 ± 1.79 ^c^	1.157 ± 3.01 ^b^	9.457 ± 1.16 ^a^	20.13 ± 2.11 ^b^	31.94 ± 8.32 ^a^	30.81 ± 3.63 ^a^	408.5 ± 12.71 ^b^
43-As	*Glebionis coronaria* (Crown Daisy)	88.00 ± 2.67 ^a^	12.56 ± 1.03 ^b^	23.39 ± 5.49 ^a^	4.10 ± 0.29 ^b^	27.23 ± 3.75 ^a^	20.16 ± 0.95 ^a^	41.75 ± 4.30 ^a^	360.6 ± 13.23 ^a^
66-As	*Centaurea nicaeensis* (Mediterranean Star-Thistle)	86.86 ± 2.04 ^a^	11.75 ± 0.69 ^b^	24.94 ± 4.83 ^a^	0.45 ± 0.92 ^b^	19.13 ± 1.63 ^b^	21.44 ± 0.14 ^a^	34.65 ± 3.50 ^a^	350.5 ± 7.26 ^b^
74-Pl	*Bellardia trixago* (Mediterranean Lineseed)	85.8 ± 0.66 ^b^	13.42 ± 0.11 ^b^	27.00 ± 0.73 ^a^	0.99 ± 1.26 ^b^	17.78 ± 3.91 ^b^	16.18 ± 7.02 ^a^	42.44 ± 3.77 ^a^	339.6 ± 4.95 ^a^
78-Pl	*Antirrhinum tortuosum* (Greater Snapdragon)	84.83 ± 3.30 ^b^	12.63 ± 0.80 ^b^	25.36 ± 7.68 ^a^	1.02 ± 1.93 ^b^	20.4 ± 1.42 ^b^	23.08 ± 2.24 ^a^	45.58 ± 2.90 ^a^	342.8 ± 12.32 ^a^
Aromatic/medicinal plants (phenolic-rich species)									
44-La	*Teucrium fruticans* (Olive-leaved Germander)	85.61 ± 2.81 ^b^	12.91 ± 0.68 ^b^	26.68 ± 6.67 ^a^	1.17 ± 1.48 ^b^	38.42 ± 6.55 ^a^	23.1 ± 0.62 ^a^	33.57 ± 1.94 ^a^	346.4 ± 11.04 ^a^
46-Ap	*Foeniculum vulgare* (Fennel)	85.51 ± 1.77 ^b^	12.47 ± 1.17 ^b^	23.02 ± 3.94 ^a^	2.57 ± 0.65 ^b^	24.41 ± 3.31 ^a^	27.32 ± 5.81 ^a^	26.53 ± 5.86 ^b^	347.9 ± 10.37 ^a^
47-La	*Prasium majus* (White Hedge-Nettle)	85.12 ± 3.32 ^a^	13.93 ± 0.18 ^a^	31.97 ± 4.87 ^a^	2.613 ± 0.51 ^b^	23.66 ± 1.52 ^b^	21.03 ± 2.64 ^a^	31.24 ± 0.56 ^a^	336.5 ± 8.49 ^b^
61-Am	*Allium subhirsutum* (Hairy Garlic)	91.79 ± 4.84 ^b^	12.21 ± 0.40 ^b^	15.65 ± 9.96 ^a^	3.36 ± 2.78 ^a^	16.65 ± 0.95 ^b^	24.84 ± 5.87 ^a^	46.56 ± 2.11 ^a^	368 ± 12.28 ^a^
68-As	*Matricaria chamomilla* (Scented Mayweed)	88.49 ± 1.85 ^a^	15.6 ± 0.07 ^a^	27.31 ± 3.13 ^a^	2.427 ± 1.18 ^b^	17.19 ± 2.53 ^b^	20.03 ± 0.75 ^a^	35.54 ± 0.39 ^a^	349.7 ± 6.14 ^a^

DM, dry matter; ASH, ash content; CP, crude protein; EE, ether extract; NDF, neutral detergent fibre; ADF, acid detergent fibre; NFC, non-fibre carbohydrate. Within each column, means sharing the same superscript letter are not significantly different according to one-way ANOVA followed by Tukey’s HSD test (*p* < 0.05).

**Table 2:** The total polyphenolic content and antioxidant activity of the thirty-two plant species.

Identifier	Latin Name (Common Name)	PolyP (% *w*/*w*)	IC_50_ (mg/mL)
4-Bo	*Borago officinalis* (Common Borage)	0.26 ± 0.01 ^d^	2.97 ± 0.61 ^d^
14-Fa	*Scorpiurus muricatus* (Many-flowered Scorpiurus)	0.52 ± 0.02 ^c^	3.50 ± 0.86 ^c^
15-Pl	*Plantago lagopus* (Mediterranean Plantain)	0.17 ± 0.02 ^e^	2.61 ± 0.24 ^d^
18-Po	*Rumex bucephalophorus* (Red Dock)	0.13 ± 0.02 ^e^	0.37 ± 0.08 ^d^
19-As	*Sonchus oleraceus* (Smooth Sow Thistle)	0.59 ± 0.06 ^b^	4.42 ± 1.15 ^c^
20-As	*Calendula arvensis* (Field Marigold)	0.30 ± 0.01 ^d^	22.73 ± 2.17 ^b^
21-As	*Reichardia picroides* (Common Reichardia)	0.30 ± 0.02 ^d^	5.30 ± 0.31 ^c^
22-As	*Sonchus asper* (Prickly Sow Thistle)	0.11 ± 0.02 ^e^	10.89 ± 1.31 ^c^
27-Ma	*Malva sylvestris* (Common Mallow)	0.11 ± 0.01 ^e^	7.22 ± 2.44 ^c^
28-Ra	*Nigella damascena* (Love-in-a-mist)	0.46 ± 0.01 ^c^	14.37 ± 1.83 ^b^
29-Ra	*Adonis microcarpa* (Pheasant’s Eye)	0.43 ± 0.02 ^c^	5.27 ± 1.21 ^c^
30-Br	*Brassica rapa subsp. sylvestris* (Wild Turnip)	0.46 ± 0.03 ^c^	6.09 ± 0.96 ^c^
31-Er	*Erica multiflora* (Mediterranean Heath)	1.30 ± 0.02 ^a^	2.71 ± 1.32 ^d^
33-Fu	*Fumaria capreolata* (White Ramping Fumitory)	0.50 ± 0.02 ^c^	55.85 ± 5.75 ^a^
36-Ir	*Gladiolus italicus* (Field Gladiolus)	0.68 ± 0.02 ^b^	11.46 ± 2.34 ^c^
39-Xa	*Asphodelus fistulosus* (Onion Weed)	0.57 ± 0.03 ^b^	12.49 ± 3.31 ^c^
40-Xa	*Asphodelus ramosus* (Branched Asphodel)	0.33 ± 0.05 ^d^	3.50 ± 0.54 ^c^
41-As	*Galactites tomentosa* (Mediterranean Thistle)	0.20 ± 0.01 ^e^	6.52 ± 3.20 ^c^
43-As	*Glebionis coronaria* (Crown Daisy)	0.28 ± 0.01 ^d^	4.00 ± 0.54 ^c^
44-La	*Teucrium fruticans* (Olive-leaved Germander)	0.46 ± 0.02 ^c^	0.63 ± 0.08 ^d^
46-Ap	*Foeniculum vulgare* (Fennel)	0.24 ± 0.03 ^d^	6.49 ± 0.31 ^c^
47-La	*Prasium majus* (White Hedge-Nettle)	0.16 ± 0.00 ^e^	1.74 ± 0.09 ^d^
48-So	*Solanum nigrum* (Black Nightshade)	0.07 ± 0.00 ^e^	3.34 ± 0.98 ^c^
54-Ap	*Daucus carota* (Wild Carrot)	0.28 ± 0.01 ^d^	2.13 ± 0.37 ^d^
55-Ac	*Acanthus mollis* (Bear’s Breech)	0.34 ± 0.03 ^d^	9.76 ± 0.28 ^c^
61-Am	*Allium subhirsutum* (Hairy Garlic)	0.41 ± 0.01 ^c^	3.95 ± 0.73 ^c^
63-Ur	*Urtica pilulifera* (Roman Nettle)	0.12 ± 0.00 ^e^	5.57 ± 0.41 ^c^
64-Ur	*Parietaria judaica* (Pellitory-of-the-wall)	0.15 ± 0.04 ^e^	1.16 ± 0.26 ^d^
66-As	*Centaurea nicaeensis* (Mediterranean Star-Thistle)	0.23 ± 0.03 ^d^	0.43 ± 0.07 ^d^
68-As	*Matricaria chamomilla* (Scented Mayweed)	0.23 ± 0.01 ^d^	4.33 ± 0.61 ^c^
74-Pl	*Bellardia trixago* (Mediterranean Lineseed)	0.66 ± 0.04 ^b^	3.76 ± 0.47 ^c^
78-Pl	*Antirrhinum tortuosum* (Greater Snapdragon)	0.30 ± 0.01 ^d^	9.65 ± 2.46 ^c^

Means within a column sharing the same superscript letter are not significantly different according to one-way ANOVA followed by Tukey’s HSD test (*p* < 0.05).

**Table 3:** Methane production over the 48 h period and B_0_ and k results.

Methane Production (L CH_4_/kg)					
Plant Substrate Number	6 h	24 h	48 h	B_0_ (L CH_4_/g)	k (1/h)
4	13.76 ± 5.27	110.42 ± 9.00	97.93 ± 12.09 ^b^	114.01	0.063
14	14.91 ± 4.28	65.25 ± 6.08	119.02 ± 22.69 ^b^	446.71	0.006
15	37.04 ± 14.49	90.47 ± 39.25	44.49 ± 9.73 ^c^	66.32	0.167
18	9.75 ± 3.92	47.01 ± 22.27	67.65 ± 24.55 ^c^	96.07	0.026
19	25.34 ± 5.80	57.22 ± 5.24	46.10 ± 8.26 ^c^	51.96	0.130
20	22.93 ± 6.04	194.82 ± 33.77	184.38 ± 4.20 ^a^	214.51	0.057
21	16.63 ± 3.60	92.65 ± 9.73	107.33 ± 27.54 ^b^	126.21	0.044
22	68.69 ± 2.89	168.79 ± 5.52	182.55 ± 5.51 ^a^	189.76	0.081
27	32.22 ± 13.05	50.57 ± 6.91	64.33 ± 25.76 ^c^	60.63	0.110
28	105.72 ± 37.86	115.01 ± 44.67	173.49 ± 47.28 ^a^	146.03	0.202
29	35.32 ± 2.89	174.87 ± 1.44	158.24 ± 27.25 ^b^	179.35	0.070
30	22.59 ± 4.21	46.10 ± 13.27	51.37 ± 14.00 ^c^	51.74	0.095
31	12.61 ± 9.53	18.23 ± 10.67	30.39 ± 14.76 ^c^	32.08	0.048
33	12.61 ± 5.81	28.09 ± 7.65	45.18 ± 7.65 ^c^	58.30	0.030
36	38.64 ± 8.38	35.78 ± 5.18	40.59 ± 2.94 ^c^	38.34	10.709
39	35.78 ± 6.18	146.54 ± 7.16	188.05 ± 2.71 ^a^	224.66	0.040
40	56.99 ± 40.31	61.00 ± 37.14	122.35 ± 2.56 ^b^	138.30	0.037
41	57.45 ± 30.23	84.51 ± 18.80	91.39 ± 13.59 ^b^	88.90	0.170
43	37.50 ± 19.37	74.07 ± 2.48	153.31 ± 29.88 ^b^	570.99	0.006
44	72.70 ± 6.09	58.02 ± 1.95	61.69 ± 4.71 ^c^	64.14	5.657
46	29.13 ± 5.57	58.82 ± 8.94	45.75 ± 5.68 ^c^	52.31	0.151
47	89.67 ± 29.91	124.30 ± 8.54	116.16 ± 7.68 ^b^	120.36	0.229
48	7.91 ± 4.16	87.61 ± 33.67	124.53 ± 23.82 ^b^	203.03	0.020
54	5.39 ± 1.29	108.02 ± 3.71	111.91 ± 6.12 ^b^	136.60	0.043
55	48.39 ± 6.39	68.91 ± 4.12	121.66 ± 3.80 ^b^	139.01	0.038
61	78.55 ± 6.03	97.58 ± 5.79	166.38 ± 45.70 ^b^	160.98	0.063
63	12.84 ± 4.60	26.03 ± 5.67	44.03 ± 10.24 ^c^	59.48	0.027
64	10.78 ± 5.13	38.30 ± 17.10	54.24 ± 13.37 ^c^	69.219	0.032
66	26.49 ± 6.12	74.76 ± 4.47	109.05 ± 7.16 ^b^	134.36	0.035
68	33.25 ± 8.10	141.38 ± 4.17	40.82 ± 6.56 ^c^	86.85	0.143
74	57.33 ± 29.29	112.37 ± 48.84	198.26 ± 21.96 ^a^	290.24	0.023
78	28.44 ± 20.84	94.26 ± 6.17	71.78 ± 12.66 ^c^	83.99	0.102

B_0_ (L CH_4_/g) denotes the maximum methane yield obtainable per gram of substrate, while k (1/h) reflects the degradation rate and conversion speed of the substrate to methane. Means sharing the same letter are not significantly different according to Tukey’s HSD test (*p* < 0.05): ^a^ highest, ^b^ intermediate, ^c^ lowest methane producers.

**Table 4:** Classification of plant species by methane production.

Category	Criteria
(1) High B_0_ and Low k	High potential yield, slow rate of production
(2) High B_0_ and High k	High potential yield, fast production rate
(3) Moderate B_0_ and High k	Moderate yield, fast rate
(4) Moderate B_0_ and Moderate/Low k	Moderate yield, slow/moderate rate

**Table 5:** The eigenvectors for the twelve parameters, the eigenvalues and variability of the first two principal components.

Parameters	Eigenvectors	
	F1	F2
DM	0.314	0.064
Ash	−0.412	−0.258
CP	−0.424	−0.114
EE	0.357	−0.058
NDF	−0.076	−0.101
ADF	0.322	−0.333
NFC	−0.024	0.591
Energy	0.486	0.182
PolyP	−0.040	0.465
IC_50_	−0.177	0.362
B_0_	−0.184	0.249
k	0.106	−0.002
Eigenvalue	3.081	2.032
Variability (%)	25.677	16.935

**Table 6:** The categorisation of the plant species within the four clusters (AHC).

Cluster	1	2	3	4
Number of objects by cluster	8	9	5	10
Sum of weights	8	9	5	10
Within-cluster variance	4.063	6.516	4.258	6.098
Minimum distance to centroid	0.427	1.025	1.019	1.178
Average distance to centroid	1.728	2.274	1.746	2.297
Maximum distance to centroid	2.630	3.370	2.563	3.013
	*Borago officinalis*	*Scorpiurus muricatus*	*Rumex bucephalophorus*	*Calendula arvensis*
	*Plantago lagopus*	*Reichardia picroides*	*Fumaria capreolata*	*Sonchus asper*
	*Sonchus oleraceus*	*Galactites tomentosa*	*Daucus carota*	*Malva sylvestris*
	*Brassica rapa*	*Solanum nigrum*	*Parietaria judaica*	*Nigella damascena*
	*Erica multiflora*	*Acanthus mollis*	*Centaurea nicaeensis*	*Adonis microcarpa*
	*Gladiolus italicus*	*Allium subhirsutum*		*Asphodelus fistulosus*
	*Teucrium fruticans*	*Urtica pilulifera*		*Asphodelus ramosus*
	*Foeniculum vulgare*	*Matricaria chamomilla*		*Prasium majus*
		*Glebionis coronaria*		*Bellardia trixago*
				*Antirrhinum tortuosum*

## Data Availability

The data presented in this study are available on request from the corresponding author. The data are not publicly available due to privacy.
